# Cholesteatoma of the concha bullosa: a case report

**DOI:** 10.1186/1752-1947-4-407

**Published:** 2010-12-17

**Authors:** Ibrahim Cukurova, Erhan Demirhan, Ilker B Arslan, Suheyla Cumurcu

**Affiliations:** 1 Department of ENT Head and Neck Surgery, Izmir Tepecik Training and Research Hospital, Turkey; 2 Department of Pathology, Izmir Tepecik Training and Research Hospital, Turkey

## Abstract

**Introduction:**

Cholesteatoma is a relatively common disease within the middle ear cavity, but rarely it manifests in the paranasal sinuses. There is, to the best of our knowledge, only one other published case of cholesteatoma inside the concha bullosa in the English language literature.

**Case presentation:**

An 81-year-old Caucasian woman was admitted to our hospital complaining of nasal obstruction, headache and diplopia. After endoscopic and radiological evaluation a transnasal endoscopic approach was chosen. The diagnosis of cholesteatoma was established by histopathological evaluation of the mass inside the concha bullosa.

**Conclusion:**

Although it is rarely seen, cholesteatoma should be considered in the differential diagnosis of slow-growing and destructive paranasal masses.

## Introduction

Concha bullosa (CB) is the pneumatization of the middle turbinate and is one of the most common variations of the sinonasal anatomy. A 14% to 53.6% frequency of CB has been reported by various studies [1]. Many pathological entities were described in the concha bullosa such as polyps, pyocele, and mycosis [2-4]. Cholesteatoma is a relatively common disease within the middle ear cavity and temporal bone, whereas cholesteatoma of the nasal and paranasal region is an exceptionally rare entity. Cholesteatoma can be seen in the frontal, ethmoid and maxillary sinuses [5-7]. However, to the best of our knowledge, there is only one other published case of cholesteatoma inside the CB in the English language literature [8].

## Case presentation

An 81-year-old Caucasian woman presented to the outpatient clinic of our hospital with nasal obstruction, headache and diplopia. She had experienced nasal obstruction for over ten years and her headache had worsened for three months. She did not have epistaxis or epiphora. She had been treated medically with antibiotics, nasal decongestants, and nasal steroids. However, her complaints were not alleviated. Her otorhinolaryngological history was unremarkable.

An endoscopic examination revealed a massively large middle turbinate on the left side. The mucosa was normal and no infection signs were detected in the nasal passage. The enlarged middle turbinate contacting the lateral nasal wall and the septum was the only endoscopic finding. Our patient has proptosis, but her ophthalmic examination revealed 20/20 visual acuity in both eyes and normal intraocular pressures.

**Figure 1:**
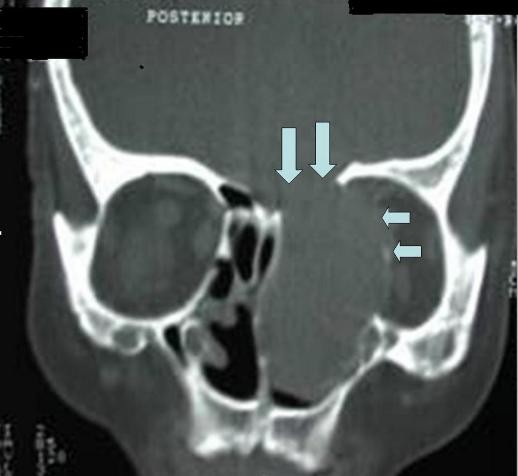
**Coronal computed tomography (CT) scan of our patient with cholesteatoma inside the concha bullosa, erosion of lamina papyracea (small arrows) and ethmoid roof (large arrows)**.

Computed tomography (CT) scans showed a homogenous fluid or soft tissue density lesion surrounded by a bony shell in the left nasal cavity. Erosion of lamina papyracea and ethmoid roof was determined from the CT scans (Figure 1). Neoplasia and mucocele of CB were considered in the differential diagnosis, and a biopsy was planned under local anesthesia.

**Figure 2:**
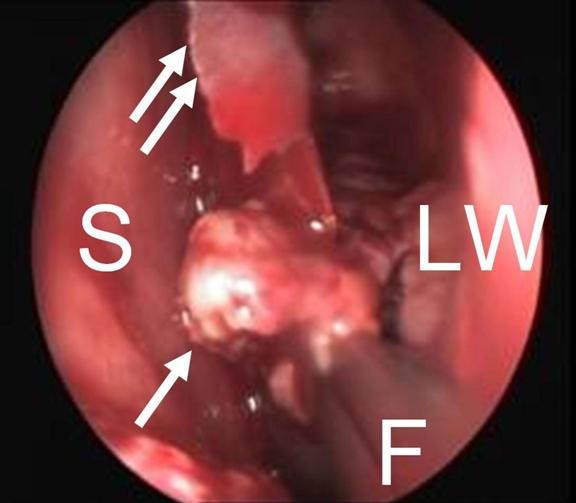
**Intraoperative view**. Double arrows, medial part of the concha bullosa; F, forceps; LW, lateral wall of the nasal cavity; S, septum; single arrow, cholesteatoma.

**Figure 3:**
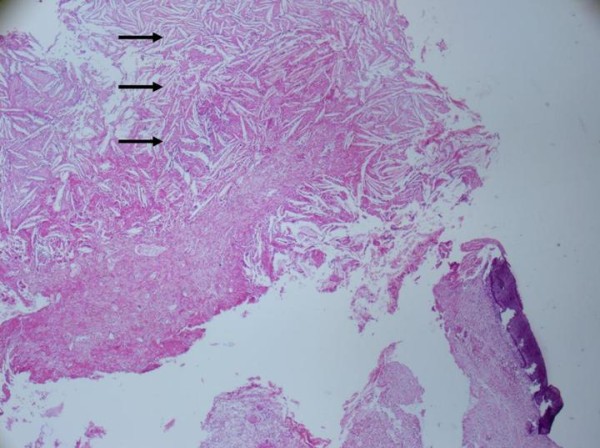
**Hisopathological section of cholesteatoma inside the concha bullosa (hematoxylin and eosin staining) submucosal chronic inflammation, cholesterol crystals (black arrows)**.

**Figure 4:**
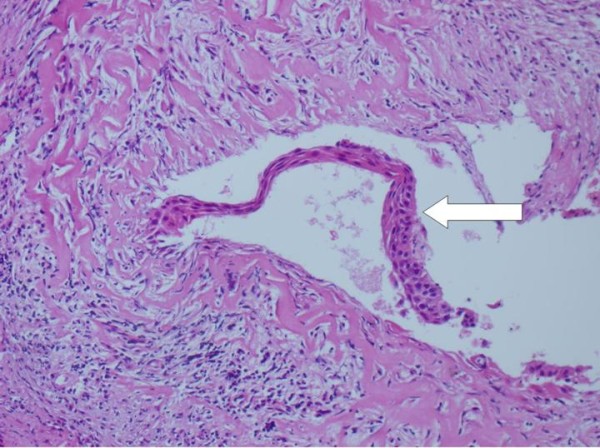
**Hisopathological section of cholesteatoma inside the concha bullosa (hematoxylin and eosin staining) squamous epithelium with keratinized debris (white arrow)**.

Intraoperatively, when we removed the lateral part of the CB for biopsy, a yellow-white colored mass was found inside the CB cavity (Figure 2). The mass was totally excised. The lamina papyracea was found to be defective but no erosion was detected at the ethmoid roof. No further treatment was performed, and the cavity was left open without packing. A histopathological examination showed submucosal chronic inflammation and squamous epithelium with keratinized debris and diagnosis of cholesteatoma was made (Figures 3 and 4). Diplopia and proptosis healed after surgery. A follow-up examination 14 months after surgery showed no recurrence of the disease.

## Discussion

Four theories on the pathogenesis of paranasal sinus cholesteatomas have been put forward [6]. The most widely accepted theory is the congenital (primary) cholesteatoma theory. Congenital (primary) cholesteatoma is believed to be a result of misplaced ectodermal, epithelial cell remnants during the formation of the face in the third to fifth week of embryogenesis [9]. The development of acquired (secondary) cholesteatomas can be explained by three theories. One theory is the implantation of epidermoid cells after surgery or trauma. Immigration and metaplasia theories are the other proposals. As in most other cases, in our patient the congenital cholesteatoma theory is the most likely explanation. The implantation theory cannot be accepted because our patient has no history of previous trauma or surgery to the head and neck region. Immigration of epithelium from the nasal vestibule to the intranasal region has never been reported and we also could not show such an intranasal tract in our case [6]. The metaplasia theory has been rejected by many authors because squamous epithelium deriving from metaplasia due to chronic rhinosinusitis is of a non-keratinizing type [6].

Most of the paranasal cholesteatoma cases were adults, as in our patient [6]. However, there are also pediatric cases in the literature [10].

Due to the rarity of this lesion, cholesteatoma in the paranasal sinuses is seldom pre-operatively diagnosed. Cholesteatoma has a slow-growing pattern and it has an erosive effect on bone. In our patient, we initially considered the possibility of a neoplastic lesion or pyocele during the differential diagnosis. However, as shown in this case, cholesteatoma can cause similar findings.

## Conclusions

Although it is rarely seen, cholesteatoma should be considered in the differential diagnosis of slow-growing and destructive paranasal masses. Total excision is the treatment of choice for paranasal cholesteatomas. Our patient was treated successfully with a transnasal endoscopic approach.

## Consent

Written informed consent was obtained from the patient for publication of this case report and any accompanying images. A copy of the written consent is available for review by the Editor-in-Chief of this journal.

## Competing interests

The authors declare that they have no competing interests.

## Authors' contributions

IC performed the surgery and supervised the writing of the manuscript. ED was the major contributor to the writing of the manuscript. IBA had a role in writing a final revision of the manuscript. SC performed the histopathological analysis and final revision of the manuscript. All authors read and approved the final manuscript.
